# Evaluation of an angio-osseo inductive bone ceramic versus demineralised freeze-dried bone allograft in the treatment of periodontal intrabony defects: a randomised controlled clinical trial

**DOI:** 10.2340/biid.v13.45748

**Published:** 2026-04-10

**Authors:** Vidya Sagar S, Uday Kiran Roopavath, Navatha Sattar, Raja Babu P, Athmuri Durga Nandini, Neema Kumari, Subha Narayan Rath

**Affiliations:** a Regenerative Stem Cells Laboratory, Department of Biomedical Engineering, Indian Institute of Technology Hyderabad, Sangareddy, India; b Department of Periodontics, Kamineni Institute of Dental Sciences, Narketpally, Nalgonda, India; c Department of Microbiology, Malla Reddy Institute of Medical Sciences, Malla Reddy Vishwavidyapeeth, Hyderabad, India

**Keywords:** allograft, β-tricalcium phosphate, alloplastic graft, ion doping, hydroxyapatite, periodontal intrabony defects

## Abstract

**Introduction:**

Periodontal intrabony defects (IBDs) respond favourably to regenerative periodontal therapy. Various graft and non-graft materials have been used, with non-allogenic bone substitutes offering the potential to enhance clinical outcomes and radiographic defect resolution. Angio-Osseo Inductive Bone Ceramic (ABC), a novel ion-doped biphasic calcium phosphate graft, has shown promise due to its osteogenic and angiogenic potential.

**Aim:**

The study aimed to compare the clinical and radiographic outcomes of ABC and demineralised freeze-dried bone allograft (DFDBA) in the surgical management of periodontal IBDs.

**Materials & methods:**

A parallel-group, examiner- and statistician-blinded, randomised controlled clinical trial was conducted on 30 IBDs (one per patient), randomly assigned to ABC (test, *n* = 15) or DFDBA (control, *n* = 15). Clinical parameters, including Plaque Index (PI), Gingival Index (GI), probing pocket depth (PPD), and clinical attachment level (CAL), were recorded at baseline, 3 months, and 6 months. Radiographic bone fill was assessed at 3 and 6 months using standardised digital radiovisiography.

**Results:**

In both groups, mean IBD scores were significantly different at all time intervals (*p* < 0.05). Intergroup differences in PPD reduction and CAL gain were not statistically significant. Radiographic analysis demonstrated significantly greater bone fill in the test group (68.63%) compared with the control group (45.48%) at 6 months (*p* < 0.05).

**Conclusion:**

Both ABC and DFDBA were effective in the treatment of periodontal IBDs. However, ABC demonstrated statistically significant improvement in defect fill, supporting its potential as a novel alloplastic alternative to conventional allograft.

## Introduction

Bone grafting is a dynamic process and has become a cornerstone of clinical practice, serving as a valuable technique in numerous reconstructive applications of modern dentistry [1]. Using bone grafts to fix bone defects has been a practice since 1923, when it was first introduced by Hegedus [2]. Most surgeons prefer bone grafts due to their demonstrated clinical efficacy, functional periodontal repair, leading to pocket reduction to a manageable level, and apparent bone defect fill [1].

Bone graft aids in regeneration through osteogenesis, osteoconduction, and osteoinduction. Commonly utilised bone grafts for treating periodontal intrabony defects (IBDs) include autografts, allografts, xenografts, and alloplastic ceramics [3].

Autogenous bone has been considered the gold standard due to its inherent osteogenic, osteoinductive, and osteoconductive properties [4]. However, its clinical application is limited due to the need for a second surgical site, the possibility of donor site morbidity, the limited amount of graft material, and the high rate of resorption after transplantation [5, 6]. To address these limitations, bone allografts, particularly demineralised freeze-dried bone graft (DFDBA), have gained popularity. The osteoinductive potential of DFDBA is due to the presence of bone morphogenic proteins (BMP) that are produced following bone demineralisation [7]. Despite these advantages, the drawbacks of allografts include the risk of host immune reaction or incompatibility, the potential for disease transmission from donor to recipient, and the potential graft contamination resulting in the recipient site [8, 9].

In response to the limitations of autografts and allografts, alloplasts (synthetic bone grafts), such as hydroxyapatite (HA) and β-tricalcium phosphate (β-TCP), have gained particular interest due to their chemical similarity to natural bone mineral, osteoconductive properties, and ability to integrate with host bone [10]. HA is a primary focus of bone graft research due to its resemblance to the inorganic part of the bone [11]. It has great chemical stability and low solubility, which are important for its osteoconductive and biocompatible qualities, both of which are essential for bone regeneration [12]. However, HA has a very slow degradation rate, which limits its utility as a standalone graft material. To address this limitation, the interest in β-TCP materials has rapidly increased in recent years. β-TCP is a resorbable ceramic that is readily replaced by new bone [13]. It also has the ability to promote angiogenesis [14] and speed up bone remodelling by making it easier for osteogenic cells to colonise [15]. Thus, the combination of β-TCP and HA results in a faster and greater rate of bone ingrowth compared to HA alone and provides better biological properties compared to β-TCP alone [16]. However, the clinical utility of these calcium phosphate-based grafts is curtailed because of their limited regenerative potential [17].

To overcome these challenges, ion doping has emerged as a promising strategy to enhance the biological activity, mechanical properties, antimicrobial effects, and solubility of this material [18]. Therefore, in this study, a novel Angio-Osseo-Inductive Bone Ceramic (ABC) graft material was developed. This graft is composed of HA and β-TCP doped with magnesium (Mg^2+^) and strontium (Sr^2+^) ions, designed to enhance the biological properties of osteogenesis and vascularisation, as well as the mechanical properties of the bone graft material. The development of such ion-doped grafts represents a significant step forward in bioengineering synthetic substitutes with autograft and allograft-like properties for effective periodontal regeneration [18, 19].

The efficacy of DFDBA in repairing human intrabony lesions has been evaluated in numerous clinical studies and has been paired with enamel matrix derivatives, platelet-rich plasma (PRP), hyaluronic acid, concentrated growth factors, and amniotic membrane [20–27]. These studies have demonstrated the superior regenerative potential of DFDBA, with promising outcomes based on both clinical and radiographic evaluations.

Despite DFDBA’s success, the development of ABC grafts represents a new frontier in periodontal regeneration. By offering synthetic grafts with enhanced bioactivity, the ABC graft may help overcome limitations related to donor tissue availability, processing inconsistencies, storage requirements, and variability in biological response.

However, there is limited clinical evidence on ion-doped biphasic calcium phosphate grafts versus established allografts in the treatment of periodontal IBD. Therefore, this study aimed to compare the effectiveness of a novel graft material, ABC (a combination of HA and β-TCP doped with Mg^2+^ and Sr^2+^ ions), with that of DFDBA for treating periodontal IBDs. Thus, the study can serve as a pioneering investigation into the comparative evaluation of these materials, providing a foundation for future clinical and translational research.

## Materials and methods

### Ethical approval, study design, and registration

This was a parallel-group interventional, randomised controlled clinical trial with blinded outcome assessment and statistical analysis. The examiner and statistician were blinded to group allocation, while the surgeon could not be blinded due to the distinct handling characteristics of the graft materials.

The study protocol was approved by the Institutional Ethics Committee of Kamineni Institute of Dental Sciences, which agreed upon the Helsinki protocols undertaken in the study (Approval No: KIDS/IEC/2020/415).

All participants provided written informed consent prior to enrolment. The trial was officially recorded in the Clinical Trials Registry of India (CTRI/2021/09/036232, dated September 3, 2021).

### Materials

The study equated ABC graft material procured from the Indian Institute of Technology in Hyderabad and DFDBA (500–1040 µm) acquired from the Mumbai tissue bank of Tata Memorial Hospital.

The exact phase ratio (HA: β-TCP) and composition of ABC are not disclosed due to a pending patent application. However, the material consists of a biphasic calcium phosphate scaffold doped with Sr^2+^ and Mg^2+^.

Briefly, the ABC graft was synthesised as a Sr^2+^ and Mg^2+^ co‑doped calcium‑deficient apatite by a wet chemical precipitation. Aqueous solutions of reagent grade calcium nitrate tetra hydrate, Ca(NO_3_)_2_•4H_2_O, strontium nitrate, Sr(NO_3_)_2_, and magnesium nitrate hexahydrate, Mg(NO_3_)_2_•6H_2_O were prepared and combined in stoichiometric proportions to obtain a calcium‑deficient apatite composition with a target Ca/*P* ratio of 1.60 and an overall (Ca+Sr+Mg)/*P* ratio of 1.80, corresponding to the ABC graft formulation. A separate diammonium hydrogen phosphate solution was prepared as the phosphate source. Wet chemical precipitation was carried out by adding the mixed cation solution to the phosphate solution under continuous stirring, maintaining the pH in the alkaline range (approximately 9) and the temperature around 60°C for apatite precipitation. The resulting Sr- and Mg-doped calcium‑deficient apatite precipitate was aged, thoroughly washed to remove residual ions, dried, and subsequently calcined at 1100°C for 2 hours to obtain crystalline ABC graft powder suitable for further processing as a bone graft material.

### Study population

Subjects were selected from outpatients, Department of Periodontics and Implantology, Kamineni Institute of Dental Sciences, Narketpally, Nalgonda (Dist.), Telangana. The study’s protocol and procedures were explained to the participants, and their written consent was obtained.

Eligible participants included systemically healthy individuals aged between 25 and 55 years with maxillary and/or mandibular IBDs. The defect characteristics chosen were Probing Pocket Depth (PPD) ≥ 5 mm, Clinical Attachment Level (CAL) ≥ 3 mm, and radiographic IBD depth (IBD) ≥ 3 mm persisting 6–8 weeks after initial periodontal therapy.

The study excluded patients with systemic diseases or medically compromised conditions, current use of medications affecting periodontal tissue, pregnant or lactating women, known allergies to graft materials or local anaesthetics, smokers and former smokers, and individuals with parafunctional habits such as bruxism.

### Sample size and randomisation

The sample size was estimated using a two-tailed *t*-test with a power (β) of 80% and α of 5%. It was established that each study group would require a minimum of 13 defects, but this was increased to 15 defects per group in order to account for any dropouts. The G Power software (version 3.1.9.7, Heinrich-Heine University, Düsseldorf, Germany) was used to calculate the sample size.

Thirty patients diagnosed with chronic periodontitis who met the inclusion and exclusion criteria were enrolled. A total of 30 IBDs (one per patient) were included in the study.

A simple randomisation technique (coin toss method) was used to assign patients into either treatment group: Test group: 15 defects were treated with ABC graft. Control group:15 defects were treated with DFDBA. Allocation was performed by a staff member not involved in outcome measurements.

### Parameters

All clinical parameters were recorded using a UNC-15 periodontal probe and documented in a standardised proforma. In this study, PI [28], GI [29], PPD, and CAL were considered as secondary clinical outcome measures, while linear measurement from the Cemento-Enamel Junction to the Bottom of the Defect (CEJ-BD) was considered as the primary endpoint of the study. Prefabricated custom acrylic stents with guiding grooves were used at all time points to ensure accuracy and reproducibility of measurements.

### Radiographic assessment of IBD

Radiovisiography (RVG) was standardised using the long cone paralleling technique. Radiographs were taken at baseline, 3 and 6 months. IBD was assessed by measuring the vertical distance from CEJ-BD, using UTHSCSA Image Tool™ software. Radiographs were standardised using a metal ball with a known diameter of 3.98 mm, which was incorporated into the RVG sensor. The actual diameter of the metal ball was confirmed using a digital vernier calliper.

The UTHSCSA software was used to calculate the difference between the actual diameter of the metallic ball and the diameter measured in the radiograph. These measurements were then used to adjust for any foreshortening or magnification that was present in the radiographs.

The following formula was used to calculate the percentage of radiographic bone fill in the study groups.


$$Radiographic\;bone\;fill\left(\%\right)=\frac{Baseline\;CEJ-BD-6\;month\;CEJ-BD}{Baseline\;CEJ-BD}\times 100$$

### Interventions

#### Pre-surgical

Following patient selection, non-surgical periodontal therapy was performed. Oral hygiene instructions were prioritised. After 6–8 weeks, reassessment was carried out to ascertain whether surgical intervention was necessary, with the presence of PPD ≥ 5 mm, CAL ≥ 3 mm, and IBD ≥ 3 mm, as determined by radiography.

#### Surgical procedure

Local anaesthesia was administered using Lignox^®^ 2% A (Indoco Remedies Limited, Mumbai, India) to anesthetise the operative area, and the flap was elevated utilising BP blade #15. Area-specific curettes (Hu-Friedy, USA) were used for thorough debridement. ABC or DFDBA was grafted into the defect, depending on the randomisation. Post-surgically, buccal and lingual flaps were approximated utilising 3-0 silk sutures (Healthium Trusilk^®^, Bangalore, Karnataka, India), and the surgical site was protected with periodontal dressing (Coe Pak^®^, GC, USA).

#### Postoperative phase

Following the surgical procedure, each patient was instructed to take systemic antibiotics (500 mg capsule of amoxicillin three times a day) for 5 days and analgesics (tablet acelofenac 100 mg twice a day) for 3 days, concomitantly twice-daily 0.2% chlorhexidine HCl mouthrinse (Clohex, Dr Reddy’s, Hyderabad, India). After the surgery, participants were instructed not to brush the surgical site or cause any trauma to it. The sutures were removed after 14 days, and the patients were then told to use a soft toothbrush. Follow-up appointments were scheduled for 1, 3, and 6 months after the surgery.

### Statistical analysis

The data were analysed using the Statistical Package for Social Sciences software 20.00 program (SPSS Inc., Chicago, IL, USA). The Kolmogorov–Smirnov test was applied to assess the normality of variables, and it was found that all parameters follow a normal distribution. The intergroup comparisons between the test and control groups were performed using the independent *t*-test, while intragroup comparisons were conducted using the paired (dependent) *t*-test. Baseline variables were analysed using Pearson’s Chi-square test. Differences were considered statistically significant at *p* < 0.05.

## Results

### Patient selection and follow-up

**Figure 1:**
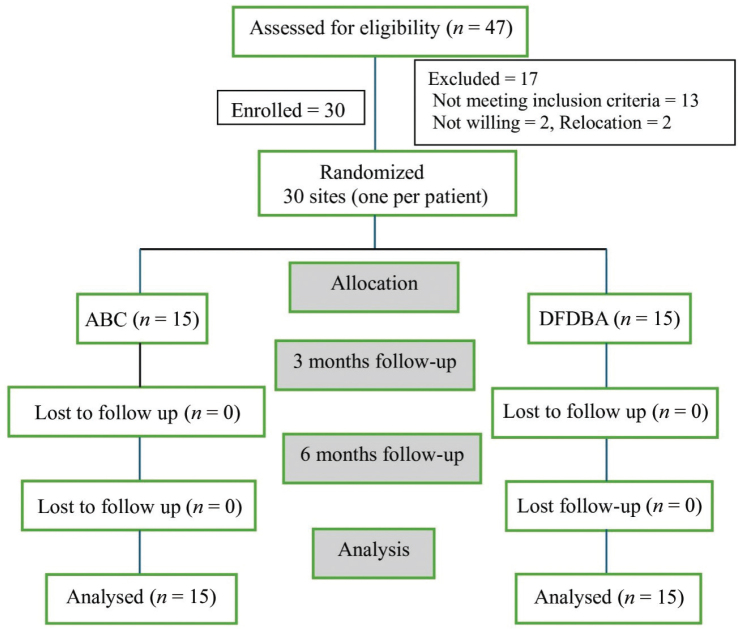
CONSORT flow diagram of patient recruitment and follow-up.

Out of 47 assessed patients, 30 were recruited. Seventeen were excluded, of which 13 did not fit into the criteria, two were not willing to participate, and two were not ready to relocate to the study site. All the recruited patients reported on time, and there was no missed follow-up (Figure 1).

### Patient characteristics

The mean age of participants in the test group was 34.6 ± 3.23 years, while in the control group, it was 36.8 ± 4.42 years. The test group consisted of seven females (46.7%) and eight males (53.3%), while the control group consisted of six females (40%) and nine males (60%). Regarding defect distribution, the test group involved eight mesial and seven distal defects, whereas the control group involved 11 mesial and 4 distal defects. Baseline characteristics between the two treatment groups were not significantly different (*p*-value > 0.05) (Table 1).

**Table 1:** Baseline characteristics of study participants.

Parameter	Test	Control	Pearson’s chi-square test *P*-value
Age (mean ± SD)	34.6 ± 3.23 years	36.8 ± 4.42 years	
Male	8 (53.3%)	9 (60%)	0.22
Female	7 (46.7%)	6 (40%)	0.20
Mesial defect	8 (33.3%)	11 (73.3%)	0.18
Distal defect	7 (66.6%)	04 (26.7%)	

### Clinical parameters

In both groups, a statistically significant reduction in mean PI and GI was observed at all time intervals (*p* < 0.05). The intergroup comparison of mean PI scores was significantly different at baseline to 3 months and baseline to 6 months (*p* < 0.05; Table 2). The intergroup comparison of mean GI scores was significantly different at baseline to 6 months (*p* < 0.05; Table 3).

**Table 2:** Comparison of PI at various time intervals.

Clinical parameter	Time intervals (months)	Test (Mean ± SD)	Control (Mean ± SD)	*P*-value^a^
PI score	Baseline	1.50 ± 0.12	1.49 ± 0.15	0.6632
	3	1.14 ± 0.18	1.24 ± 0.14	0.2540
	6	0.90 ± 0.17	1.00 ± 0.18	0.2211
	Baseline–3M	0.36 ± 0.10	0.25 ± 0.07	0.0032*
	3M–6M	0.24 ± 0.03	0.24 ± 0.08	0.8846
	Baseline–6M	0.60 ± 0.10	0.49 ± 0.14	0.0251*
*P*-value^b^		0.0007*	0.0007*	

Note:

a Independent *t*-test was used to compare the groups at different time intervals.

b Paired *t*-test to compare within the group between time intervals. PI: Plaque Index.

* indicates signficant different, *p*<0.05.

**Table 3:** Comparison of the GI at various time intervals.

Clinical parameter	Time intervals (months)	Test (Mean ± SD)	Control (Mean ± SD)	*P*-value^a^
GI score	BL	1.36 ± 0.13	1.24 ± 0.23	0.0971
	3M	1.09 ± 0.15	1.02 ± 0.23	0.3297
	6M	0.85 ± 0.17	0.84 ± 0.23	0.9174
	BL–3M	0.27 ± 0.09	0.22 ± 0.07	0.1647
	3M–6M	0.24 ± 0.02	0.18 ± 0.08	0.0500
	BL–6M	0.52 ± 0.10	0.40 ± 0.12	0.0128*
*P*-value^b^		0.0007*	0.0007*	

Note:

a Independent *t*-test was used to compare the groups at different time intervals.

b Paired *t*-test to compare within the group between time intervals.

GI: Gingival Index.

**Figure 2:**
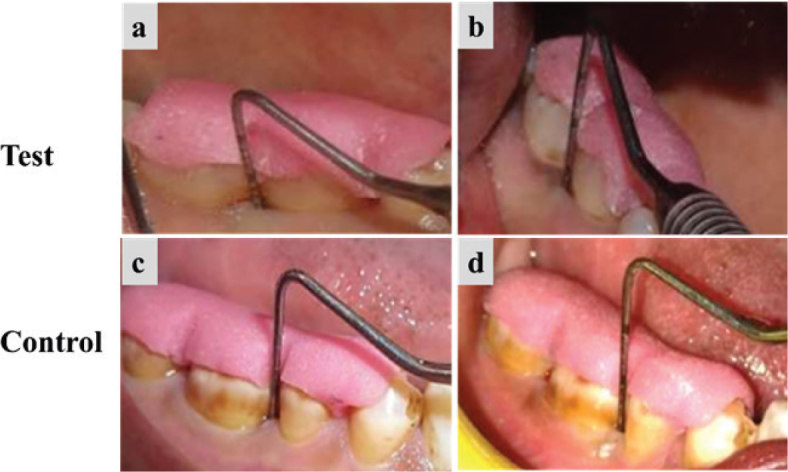
Clinical evaluation of the test group and the control group. (a) and (c) Measurement of PPD at baseline, (b) and (d) Measurement of PPD at 6 months. PPD: probing pocket depth.

Both groups demonstrated significant (*p* < 0.05) improvement in CAL gain and PPD reduction at 3 and 6 months postoperatively. The intergroup comparison revealed greater PPD reduction (Figure 2 and Table 4) and CAL gain in the test group (Table 5) but not statistically significant at any time points (*p* > 0.05).

**Table 4:** Comparison of PPD at various time periods.

Clinical parameter	Timepoints (months)	Test (Mean ± SD)	Control (Mean ± SD)	*P*-value^a^
PPD (mm)	BL	7.60 ± 2.38	7.20 ± 1.78	0.6068
	3M	4.93 ± 1.16	4.93 ± 1.28	1.0000
	6M	3.07 ± 0.88	3.27 ± 1.03	0.5733
	Δ BL–3M	2.67 ± 1.54	2.27 ± 0.70	0.3688
	Δ 3–6M	1.87 ± 0.74	1.67 ± 0.49	0.3910
	BL–6M	4.53 ± 1.77	3.93 ± 0.96	0.2578
*P*-value^b^		0.0001*	0.0001*	

Note:

a Independent *t*-test was used to compare the groups at different time intervals.

b Paired *t*-test to compare within the group between time intervals.

PPD: probing pocket depth.

**Table 5:** Comparison of CAL at various time periods.

Clinical parameter	Timepoints (months)	Test (Mean ± SD)	Control (Mean ± SD)	*P*-value^a^
CAL (mm)	BL	7.07 ± 2.05	6.63 ± 1.27	0.4929
	3M	3.80 ± 1.15	3.53 ± 1.48	0.5858
	6M	2.80 ± 0.77	2.70 ± 1.00	0.7612
	Δ BL–3M	3.27 ± 1.16	3.10 ± 0.91	0.6654
	Δ 3–6M	1.00 ± 0.65	0.83 ± 0.62	0.4791
	Δ BL–6M	4.27 ± 1.62	3.93 ± 0.75	0.4768
*P*-value^b^		0.0001*	0.0001*	

Note:

a Independent *t*-test was used to compare the groups at different time intervals.

b Paired *t*-test to compare within the group between time intervals.

CAL: clinical attachment level.

### Radiographic parameters

**Figure 3:**
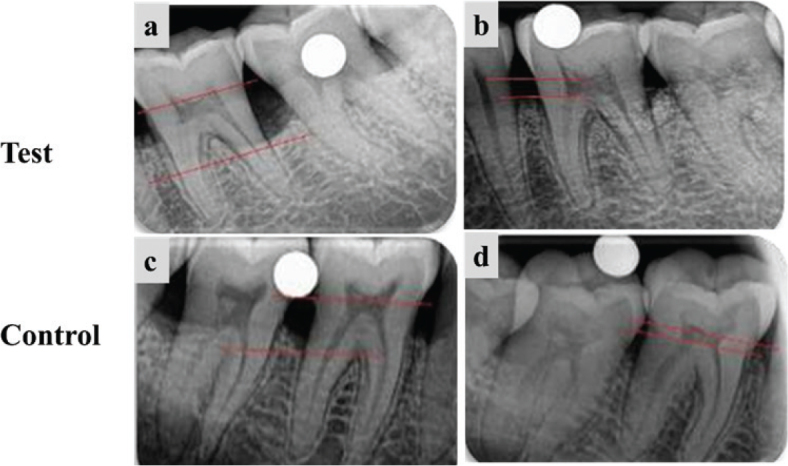
Radiographic evaluation of the test group and the control group. (a) and (c) Measurement of IBD at baseline, (b) and (d) Measurement of IBD fill at 6 months. IBD: intrabony defects.

In both groups, mean IBD scores were significantly different at all time intervals (*p* < 0.05). The intergroup comparison of the mean IBD score was significantly different at 3 months (*p* = 0.002) and 6 months (*p* = 0.002), though baseline values did not vary significantly. Similarly, the difference in values from baseline to 3 months (*p* = 0.0007) and baseline to 6 months (*p* = 0.004) was significantly different between the two groups (Figure 3 and Table 6).

**Table 6:** Comparison of IBD at various time periods.

Clinical parameter	Timepoints (months)	Test (Mean ± SD)	Control (Mean ± SD)	*P*-value^a^
IBD depth (mm)	BL	7.46 ± 2.54	7.08 ± 1.56	0.6179
	3M	3.22 ± 1.72	4.89 ± 0.87	0.0024*
	6M	2.34 ± 1.33	3.86 ± 1.09	0.0020*
	Δ BL–3M	4.24 ± 1.67	2.19 ± 1.24	0.0007*
	Δ 3–6M	0.88 ± 0.75	1.03 ± 1.13	0.6713
	Δ BL–6M	5.12 ± 1.64	3.22 ± 1.68	0.0040*
*P*-value^b^		< 0.05	< 0.05	

Note:

a Independent *t*-test was used to compare the groups at different time intervals.

b Paired *t*-test to compare within the group between time intervals.

IBD: intrabony defects.

Bone fill in the DFDBA group was 45.48%, and in ABC it was 68.63%. This intergroup difference was clinically and statistically significant (*p* < 0.05).

## Discussion

Bone replacement grafts have long been utilised in periodontal regeneration to support new bone formation and promote healing of osseous defects [30]. While autografts and allografts are considered standards, their limitations have necessitated the exploration of alternative synthetic materials. In this study, the potential role of ABC, a novel graft material composed of HA and β-TCP doped with Mg^2+^ and Sr^2+^ ions, was evaluated in IBD treatment.

Several metal ions, including Mg^2+^, Sr^2+^, copper, zinc, silver, iron, cobalt, boron, and zirconium, have been shown to play a role in tissue regeneration, and their incorporation into calcium phosphate-based grafts has demonstrated improved outcomes in bone and periodontal regeneration in animal and *in vitro* studies [19].

Mg^2+^, the fourth most abundant cation in the human body [31], is primarily found around the hydrated layers of bone apatite [32, 33]. It plays a vital role in osteogenesis and angiogenesis. It promotes the expression of Hypoxia-Inducible Factor-1α (HIF-1α) and activates peroxisome proliferator-activated receptor gamma coactivator-1α (PGC-1α), thereby enhancing mitochondrial biogenesis and osteogenic differentiation [34–36].

In this study, these two bivalent ions (Mg^2+^ and Sr^2+^) in combination were used, which amplifies the individual biological and mechanical benefits, providing a synergistic enhancement of calcium phosphate-based materials [37].

Mg^2+^ induces endothelial nitric oxide synthase (eNOS) in endothelial cells to induce angiogenesis [38]. Mg^2+^ promotes the release of calcitonin gene-related peptide (CGRP) via sensory neurons, enhancing the osteogenic potential of periodontal ligament stem cells (PDLSCs) [39–42].

Sr^2+^, a critical trace element [43], promotes bone formation and inhibits resorption through a dual mechanism [44]. Due to its chemical similarity to calcium, Sr^2+^ binds to the calcium-sensing receptor (CaSR), expressed on osteoblasts, activating intracellular signalling pathways including Wnt/β-catenin, NFATc/Maf, PI3K/Akt, COX-2/PGE₂, and MAPK (Ras/Raf/ERK) [45].

These pathways enhance the expression of key osteogenic transcription factors and matrix proteins such as Runx2, alkaline phosphatase, bone sialoprotein, and osteocalcin, and promote osteoblast proliferation and differentiation [46–49].

Sr^2+^ also suppresses PPAR-γ2, directing mesenchymal stem cell differentiation away from adipogenesis towards osteoblastogenesis [50, 51]. Sr^2+^ indirectly inhibits osteoclastogenesis by increasing osteoprotegerin (OPG) and decreasing RANKL expression in osteoblasts, thereby reducing the RANKL/OPG ratio and suppressing osteoclast precursor differentiation [52]. It also directly induces apoptosis in mature osteoclasts through CaSR-mediated activation of PLCβ, protein kinase C (PKC), and NF-κB signalling pathways. These effects result in reduced bone resorption and osteoclast survival [53, 54]. However, since these biological mechanisms are supported by experimental and preclinical studies, their direct clinical translation should be done with caution.

Mg^2+^ and Sr^2+^ doped HA and β-TCP have demonstrated enhanced osteogenesis, angiogenesis, and improved solubility, mechanical strength, bioactivity, and antimicrobial properties [18, 19]. These advancements make such composites highly promising for bone and periodontal regeneration, representing a next-generation approach in regenerative dentistry and forming the basis for this research.

The primary outcome of the study included the radiographic bone defect fill, and the secondary outcomes included change in PI, GI, PPD, and CAL. No uneventful healing and postoperative complications were observed in any of the studied groups.

Both groups revealed a significant difference and change in their mean PI and GI scores over time and throughout the study period, respectively. This outcome can be attributed to the strict oral hygiene regimen, consistent follow-up visits, and repeated instruction on oral hygiene provided to the participants. Therefore, PI and GI outcomes should not be attributed to graft performance.

The intergroup comparison of mean PI scores revealed a statistically significant difference from baseline to 3 months and from baseline to 6 months. Similarly, the mean GI scores showed a significant intergroup difference from baseline to 6 months.

Both the regenerative approaches had similar effects on the outcomes of PPD and CAL, with numerically greater mean changes in the test group, which, though, was not statistically significant.

The mean PPD reduction at 6 months with the ABC graft was 4.53 mm, which is consistent with outcomes reported using various bone graft materials. Bozic et al. reported a 4.54 mm decrease with a combination of hyaluronic acid and deproteinised porcine bone mineral, and Jalaluddin et al. observed a 4.00 mm reduction using Ossifi^®^, a biphasic HA-β-TCP ceramic with PRP [55, 56].

Similarly, some studies reported reductions in PPD, ranging from 4.08 to 4.5 mm, aligning with the results of this study [57, 58]. Rosen et al. reported a slightly higher PPD reduction of 4.8 mm using FDBA with recombinant platelet-derived growth factor-BB, while Rajesh et al. reported greater reductions, exceeding 5 mm, using calcium phosphate cements (CPC) [59, 60].

The study also found a mean CAL gain of 4.27 mm after 6 months, which aligns with findings from Jalaluddin et al., who reported a gain between 4.0 and 4.17 mm [56]. Rajesh et al. found a higher CAL gain of 5.15 mm with CPC, while Khosropanah et al. observed an increase of 3.6 mm using DFDBA and PRF [58, 60]. These results indicate that the CAL gains from ABC treatment are within the typical range reported for various bone graft materials.

In terms of radiographic bone fill, the study reported a mean fill of 5.12 mm after 6 months, which was greater than those documented in earlier research. Aspriello et al. reported a bone fill of 3.5 mm [23], and Khosropanah et al. reported radiographic defect fill of 2.1 mm [58].

Bhatia et al. reported 56.91% of defect fill in the HA group, and PRP+ HA had 57.16% [61]. In this study, bone fill in the DFDBA group is 45.48%, whereas in the doped biphasic ceramic resulted in 68.63%, which was higher than that of the control group. In addition, the observed advantage of ABC was primarily radiographic, as there was no significant difference between the two groups with regard to PPD reduction and CAL gain.

The findings of this study suggest that the novel ABC graft may represent a next-generation bone graft material with regenerative potential of autografts and allografts, offering a favourable combination of biocompatibility, osteoinductive, osteoconductive, and pro-angiogenic properties. Furthermore, it may serve as a viable regenerative alternative, especially in cases where autografts or allografts are unavailable or cost-prohibitive.

However, there are certain limitations of this study. Firstly, the study did not use advanced diagnostic aids such as CBCT for assessment. Secondly, the bone biomarkers specific for bone remodelling were not studied. Thirdly, the measurement of bone fill was done for only a short duration (6 months) of postoperative healing. Fourthly, it was a single-centred study. Fifthly, the coin toss method did not include allocation concealment, which may introduce selection bias.

Therefore, future studies should focus on patients from multiple centres, with a larger sample size, advanced diagnostic aids, and longer duration to provide more clinical evidence for the regular use of this material.

## Conclusion

Selecting the optimal grafting biomaterial can be challenging for clinicians due to the wide array of options available. In this study, the ABC graft showed a significantly higher radiographic bone fill than that of DFDBA. PPD reduction and CAL gain remained non-significant between the groups. These findings suggest that the ABC grafting material has regeneration potential and bone fill capability in periodontal IBDs compared to DFDBA. Furthermore, multicentre studies with larger sample sizes are needed to provide more robust clinical evidence to take advantage of its better properties and support its widespread use.

## Data Availability

The data supporting this study’s findings are available from the corresponding author upon reasonable request.
